# High-grade ovarian serous carcinoma patients exhibit profound alterations in lipid metabolism

**DOI:** 10.18632/oncotarget.22076

**Published:** 2017-10-26

**Authors:** Elena Ioana Braicu, Silvia Darb-Esfahani, Wolfgang D. Schmitt, Kaisa M. Koistinen, Laura Heiskanen, Päivi Pöhö, Jan Budczies, Marc Kuhberg, Manfred Dietel, Christian Frezza, Carsten Denkert, Jalid Sehouli, Mika Hilvo

**Affiliations:** ^1^ Department of Gynecology, Charité – Universitätsmedizin Berlin, Corporate Member of Freie Universität Berlin, Humboldt-Universität zu Berlin, and Berlin Institute of Health, Berlin, Germany; ^2^ On Behalf of the Tumor Bank Ovarian Cancer Network, Berlin, Germany; ^3^ Institute of Pathology, Charité – Universitätsmedizin Berlin, Corporate Member of Freie Universität Berlin, Humboldt-Universität zu Berlin, and Berlin Institute of Health, Tumor bank Ovarian Cancer Network, Berlin, Germany; ^4^ Zora Biosciences Oy, Espoo, Finland; ^5^ VTT Technical Research Centre of Finland, Espoo, Finland; ^6^ Division of Pharmaceutical Chemistry and Technology, Faculty of Pharmacy, University of Helsinki, Helsinki, Finland; ^7^ MRC Cancer Unit, Hutchison/MRC Research Centre, University of Cambridge, Cambridge, UK

**Keywords:** lipidomics, biomarker, diagnosis, prognosis, ovarian cancer

## Abstract

Ovarian cancer is a very severe type of disease with poor prognosis. Treatment of ovarian cancer is challenging because of the lack of tests for early detection and effective therapeutic targets. Thus, new biomarkers are needed for both diagnostics and better understanding of the cellular processes of the disease. Small molecules, consisting of metabolites or lipids, have shown emerging potential for ovarian cancer diagnostics. Here we performed comprehensive lipidomic profiling of serum and tumor tissue samples from high-grade serous ovarian cancer patients to find lipids that were altered due to cancer and also associated with progression of the disease. Ovarian cancer patients exhibited an overall reduction of most lipid classes in their serum as compared to a control group. Despite the overall reduction, there were also specific lipids showing elevation, and especially alterations in ceramide and triacylglycerol lipid species were dependent on their fatty acyl side chain composition. Several lipids showed progressive alterations in patients with more advanced disease and poorer overall survival, and outperformed CA-125 as prognostic markers. The abundance of many serum lipids correlated with their abundance in tumor tissue samples. Furthermore, we found a negative correlation of serum lipids with 3-hydroxybutyric acid, suggesting an association between decreased lipid levels and fatty acid oxidation. In conclusion, here we present a comprehensive analysis of lipid metabolism alterations in ovarian cancer patients, with clinical implications.

## INTRODUCTION

One of the most fundamental differences between cancer and non-malignant cells is their metabolism [1]. Besides the most established rewiring of central carbon metabolism, cancer cells exhibit alterations in lipid metabolism. Indeed, most solid tumors increase *de novo* synthesis of fatty acids to sustain the demand of membrane lipids in rapidly proliferating malignant cells [2]. Other types of tumors, including ovarian cancer, increase the utilization and oxidation of exogenous fatty acids as energy source [3]. This peculiar metabolic reprogramming is supported by the observation that ketone bodies and acyl carnitines are elevated in tumor and/or serum samples from ovarian cancer patients [4, 5].

Ovarian cancer is characterized by a very poor prognosis, mostly because the disease is detected at a late stage, leaving only limited therapeutic opportunities. Since patients with stage I disease have significantly better survival rate than patients with stage III or IV ovarian cancer, early diagnosis is critical [6]. The metabolic alterations of ovarian tumors has prompted several metabolomics and lipidomics studies to investigate the diagnostic potential of small molecules in body fluids. One of the first metabolomic studies on ovarian cancer was performed using Nuclear Magnetic Resonance (NMR). This analysis showed a good separation of serum samples from epithelial ovarian cancer patients from healthy controls or patients with benign ovarian cysts [7]. Subsequently, LC-MS profiling revealed that metabolite profiles not only distinguish patients from controls, but can also separate early-stage patients from late-stage patients [5]. A more recent study found that 16 small molecules, including many lipids, can distinguish patients with serous ovarian carcinomas from normal healthy controls [8].

To further improve the strength of this approach, we recently performed a comprehensive metabolomics analyses of blood and tissue samples from high-grade serous ovarian cancer patients and found that hydroxybutyric acids can be used both as diagnostic and prognostic biomarkers [9]. Here, we performed untargeted lipidomic profiling from the same study cohort to fully assess the changes in lipid metabolism in these patients and to find novel small molecule biomarkers.

## RESULTS

### Ovarian cancer patients exhibit decreased serum levels of distinct lipid classes

**Figure 1:**
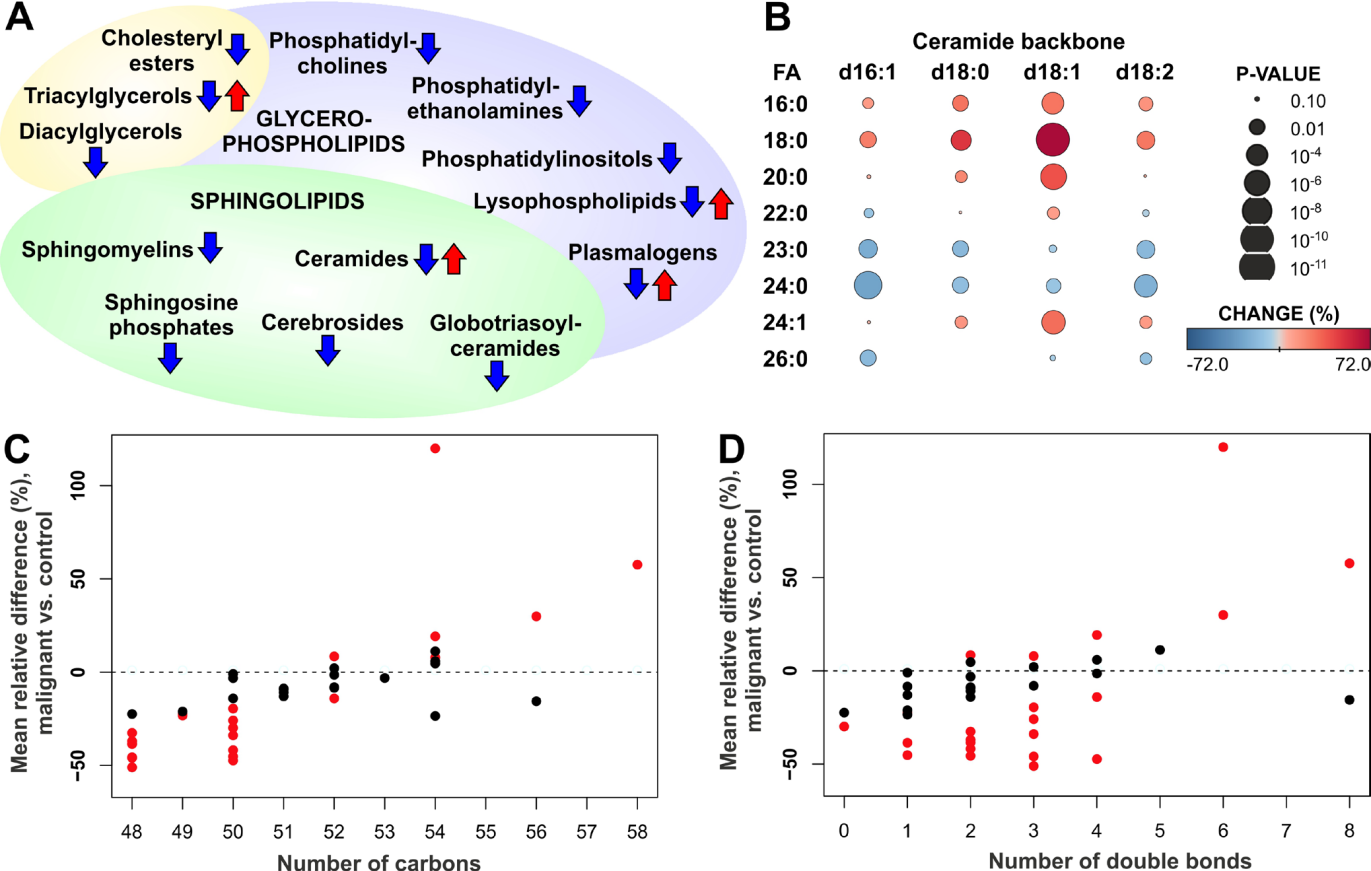
Alteration of serum lipids in ovarian cancer patients (**A**) Summary of increased and decreased lipid classes in ovarian cancer patients. (**B**) Heatmap showing increase and decrease of ceramide species with different backbone (d16:1, d18:0, d18:1, d18:2) and fatty acyl (FA) side chains in ovarian cancer patients vs. control subjects. (**C**) Mean relative change of triacylglycerol (TAG) lipids according to the total number of carbons in the FA side chains. (**D**) Mean relative change of TAG lipids according to the total number of double bonds in the FA side chains. In panels C and D red color indicates statistically significant (*p* < 0.05) result.

To investigate the alterations in the lipid profile of ovarian high-grade serous carcinoma patients, we performed a comprehensive lipidomic analysis of serum samples from 147 ovarian cancer patients and 98 control subjects with benign ovarian tumors and non-neoplastic diseases (Supplementary Table 1). In ovarian cancer patient samples a consistent decrease of most of the analyzed lipid classes was observed, including phosphatidylcholines (PCs), phosphatidylethanolamines (PEs), phosphatidylinositols (PIs), cholesterylesters (CEs), diacylglycerols (DAGs), sphingomyelins (SMs), cerebrosides (glucosyl/galactosylceramides (Glc/GalCers), lactosylceramides (LacCers)), globotriasoylceramides (Gb3s) and sphingosine-1-phosphates (S1Ps) (Figure 1A, Supplementary Table 2). To exclude the possibility that patient age could explain the observed lipid alterations, we also calculated age-adjusted *p-*values for the comparison of ovarian cancer patients vs. control subjects. The results confirmed that most of the lipids associated with malignancy remained significant after adjustment with age (Supplementary Table 2).

Lysophospholipids, including lysophosphatidylcholines (LPCs) and lysophosphatidylethanolamines (LPEs), as well as ceramides (Cers), triacylglycerols (TAGs) and plasmalogens showed variable trend depending on the specific lipids of these lipid classes. In particular, the response of ceramide species was dependent on the fatty acyl (FA) side chain composition: all the analyzed ceramides, including those with d16:1, d18:0, d18:1 and d18:2 backbones, with 16:0, 18:0, 20:0 and 24:1 FAs were increased, while those containing 23:0 and 24:0 FAs were decreased (Figure 1B). This phenomenon was particularly prominent for the most abundant d18:1 ceramides. The trend of TAG lipid species was also dependent on the FA side chains, as lipids with short FA side chains were decreased, whereas long chain TAGs were at the same level or increased in the serum of ovarian cancer patients as compared to control subjects (Figure 1C). The trend was less apparent with respect to TAG FA saturation level, although TAGs with 6 or 8 double bonds showed high elevation in ovarian cancer patients (Figure 1D). No trend was observed for DAG lipid species (Supplementary Figure 1).

We then investigated whether lipids can improve the predictive value of diagnosis of malignancy, alone or in combination with CA-125, the cancer biomarker currently used in the clinic [6]. CA-125 showed very high AUC value of 0.968 in this dataset, and none of the lipids outperformed this (Supplementary Table 2). However, slight improvement was observed when lipids were combined together with CA-125 in a logistic regression model, and the best AUC of 0.981 was reached with combination of CA-125 and PE O-36:1. Also, combination of CA-125 with other lipids, including e.g. several plasmalogens, Glc/GalCers and lysophospholipids, outperformed CA-125 alone (Supplementary Table 2).

### Distinct lipid species are associated with progression of the disease

**Figure 2:**
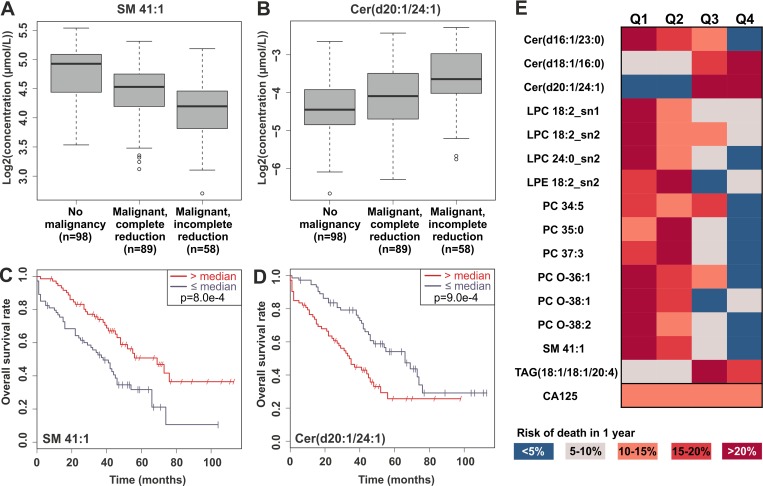
Examples of significant lipids in tumor reduction and overall survival Panels (**A**) and (**B**) present the levels of SM 41:1 and Cer(d20:1/24:1) in control subjects and in patients with partial or complete tumor reduction during the surgery, and panels (**C**) and (**D**) Kaplan-Meier curves for overall survival for the same lipids. (**E**) Heatmap demonstrating the risk of death within 1 year, when the patients have been split into quartiles based on the top-ranking lipids for overall survival or CA-125.

We then assessed which of the lipids could be used as markers of disease progression. First, we investigated which lipids were associated with either complete or incomplete tumor removal during the surgery, in terms of macroscopic residual mass. Lipids that were statistically significantly altered in patients with malignant disease and showed consistent direction of change when comparing patients with incomplete vs. complete tumor reduction are shown in Supplementary Table 3. In particular, lipids belonging to the CE, SM, LPC, PC, PC O and PE O lipid classes were decreased in all ovarian cancer patients, and progressed to lower levels especially in patients where the whole macroscopic tumor could not be removed during the surgery, as illustrated for one lipid, SM 41:1, in Figure 2A. Ceramide species showed consistent behavior, i.e. ceramides elevated in cancer patients continued to increase during disease progression (Figure 2B, Supplementary Table 3).

Second, we investigated which lipids were associated with overall and progression-free survival using cox regression models and log-rank test with median split. Most of the lipids that were decreased in ovarian cancer patients were also found in lower levels in patients with worse overall survival, and the opposite phenomenon was observed only for some of the ceramide and TAG species. Table 1 presents lipids that were significantly altered in patients with malignant disease and showed consistent and significant result in adjusted (with age and success of tumor reduction) cox regression models and log-rank test. This lipid panel consisted especially of LPC, ceramide, PC and PC-O lipid classes. Example Kaplan-Meier plots are presented for SM 41:1 and Cer(d20:1/24:1) lipids (Figure 2C, 2D). The most significant lipids and CA-125 are visualized in Figure 2E; when lipids in all the cancer patients are divided into quartiles, in many cases either in the highest or lowest quartile the risk of death within 1-year follow-up is less than 5%, whereas in the quartile of opposite end more than 20%. In contrast, CA-125 did not show any prognostic value. Several LPC and PC lipid species remained significant, when the cox model was adjusted additionally with FIGO stage (Supplementary Table 4). Only occasional lipids showed significant result for progression-free survival, but it is noteworthy that Cer(d18:1/16:0) showed significant hazard ratio both in overall and progression-free survival analyses (Supplementary Table 2).

**Table 1:** Overall survival results for lipids that were significant both in the overall survival and malignant vs. benign analyses

Lipid name	Lipid class	Cox regression				Log-rank
		UHR (95% CI)	*p*-value	MHR (95% CI)	*p*-value	*p*-value
CE 14:1	CE	0.77 (0.61, 0.98)	0.033	0.77 (0.60, 0.99)	0.038	0.020
CE 17:0	CE	0.77 (0.63, 0.95)	0.013	0.80 (0.64, 0.99)	0.043	0.005
CE 22:3	CE	0.75 (0.59, 0.94)	0.012	0.79 (0.63, 0.98)	0.034	0.015
Cer(d16:1/23:0)	Cer d16:1	0.76 (0.61, 0.95)	0.015	0.78 (0.62, 0.97)	0.028	0.020
Cer(d18:1/16:0)	Cer d18:1	1.47 (1.15, 1.87)	0.002	1.36 (1.06, 1.75)	0.014	0.001
Cer(d20:1/24:1)	Cer d20:1	1.54 (1.21, 1.97)	0.001	1.32 (1.03, 1.71)	0.031	0.001
LPC 14:0_sn1	LPC	0.72 (0.57, 0.90)	0.004	0.79 (0.62, 1.00)	0.046	0.029
LPC 18:2_sn1	LPC	0.67 (0.52, 0.84)	0.001	0.75 (0.58, 0.97)	0.027	0.007
LPC 18:2_sn2	LPC	0.67 (0.53, 0.85)	0.001	0.75 (0.58, 0.96)	0.021	0.013
LPC 20:0_sn2	LPC	0.69 (0.54, 0.88)	0.003	0.76 (0.59, 0.98)	0.035	0.005
LPC 20:2_sn1	LPC	0.73 (0.58, 0.91)	0.004	0.78 (0.62, 0.98)	0.031	0.000
LPC 20:2_sn2	LPC	0.72 (0.58, 0.90)	0.004	0.78 (0.62, 0.98)	0.031	0.014
LPC 22:0_sn1	LPC	0.70 (0.55, 0.89)	0.004	0.78 (0.61, 0.99)	0.044	0.000
LPC 24:0_sn2	LPC	0.65 (0.50, 0.84)	0.001	0.70 (0.54, 0.91)	0.008	0.021
LPE 18:2_sn2	LPE	0.68 (0.53, 0.88)	0.004	0.72 (0.56, 0.94)	0.016	0.023
PC 34:3b	PC	0.74 (0.59, 0.94)	0.014	0.79 (0.63, 0.99)	0.042	0.032
PC 34:5	PC	0.67 (0.51, 0.88)	0.004	0.68 (0.52, 0.90)	0.006	0.025
PC 35:0	PC	0.74 (0.59, 0.92)	0.008	0.74 (0.59, 0.92)	0.007	0.004
PC 35:2b	PC	0.79 (0.63, 1.00)	0.049	0.79 (0.63, 0.98)	0.035	0.038
PC 35:3a	PC	0.75 (0.59, 0.96)	0.024	0.79 (0.62, 1.00)	0.047	0.033
PC 37:3	PC	0.74 (0.60, 0.93)	0.009	0.79 (0.64, 0.97)	0.025	0.002
PC 38:6a	PC	0.73 (0.58, 0.92)	0.007	0.80 (0.64, 1.00)	0.046	0.001
PC O-36:1	PC O	0.76 (0.62, 0.94)	0.011	0.78 (0.63, 0.97)	0.028	0.047
PC O-38:1	PC O	0.71 (0.57, 0.88)	0.002	0.77 (0.63, 0.96)	0.017	0.001
PC O-38:2	PC O	0.65 (0.51, 0.82)	0.000	0.70 (0.56, 0.88)	0.002	0.000
PI 32:0	PI	0.70 (0.51, 0.98)	0.037	0.70 (0.50, 0.98)	0.036	0.009
SM 41:1	SM	0.70 (0.58, 0.86)	0.001	0.78 (0.63, 0.96)	0.019	0.001
TAG(18:1/18:1/20:4)	TAG	1.38 (1.10, 1.74)	0.006	1.32 (1.03, 1.69)	0.026	0.001
CA-125	Clinical	1.24 (0.90, 1.71)	0.190	1.12 (0.80, 1.57)	0.510	0.590

### Effect on general nutritional and health status on lipidomic profiles

Given the important role of diet and nutritional state in regulating lipid profiles, we investigated whether the observed lipidomic changes were correlated with nutritional parameters. Data (nutritional intake, BMI and weight alteration) were available for 26 patients. The lipids associated with ovarian cancer survival did not correlate with BMI, while some were negatively associated with weight change of the cancer patients (Supplementary Table 2), suggesting that the nutritional status did not have major effect on the lipidomic results. Most of the lipids decreased in cancer patients were not associated with patient-reported food intake, also supporting that the overall lipidomic alterations are not associated with nutritional status of the patients. However, many of the survival-associated lipids, especially SMs and LPCs, correlated with the patient-reported food intake. Based on the present data it is not possible to conclude the direction of causality, i.e. whether lipids correlating with the severity of the disease affect appetite or whether poorer nutrition intake affects survival and the concentration of these lipids.

### Most of tumor tissue lipids do not associate with overall survival

We also analyzed lipid profiles of tumor samples and investigated their association with overall and progression-free survival. In tumor tissue analyses we used a lipidomic platform with more restricted number of lipids analyzed. First, it was noted that the total sum of triacylglycerols was considerably lower in those tumor samples obtained from ovaries than from intestine and peritoneum (Supplementary Figure 2). Inclusion of all the samples would have caused a bias in the analysis, and for this reason the survival analyses were performed only for those patients whose tumor tissue samples were obtained from ovaries. The results showed that majority of the lipids were not significant in the cox regression analysis (Supplementary Table 5), suggesting that lipid levels measured from tumor tissues cannot be used to predict survival of the patients.

### Concentration of sphingomyelins correlate between serum and tumor tissue samples

**Figure 3:**
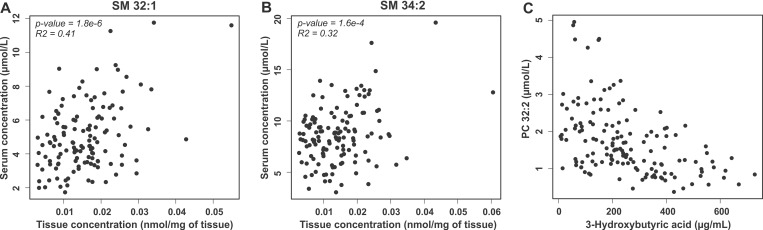
Correlation of lipids in serum and tumor tissue and with 3-hydroxybutyric acid Correlation of SM 32:1 (**A**) and SM 34:1 (**B**) concentration in serum and tumor tissue samples of ovarian cancer patients. (**C**) Correlation of PC 32:2 and 3-hydroxybutyric acid in serum samples of ovarian cancer patients.

We then analyzed the correlation of lipid distribution between serum and tumor tissue. To this aim lipidomic datasets were aligned and Pearson correlation coefficient was determined for each lipid. However, due to challenges in the alignment of the two datasets from different matrices and methodological differences, all serum lipids could not be explicitly matched with tumor tissue samples. Nevertheless, it appeared that lipids from the PC, SM and TAG lipid classes follow each other in tumor tissue most significantly (Supplementary Table 6). Notably, among the four most correlated lipids there were two SMs, SM 32:1 and SM 34:2 that are presented in Figures 3A and 3B.

### TAGs and PCs correlate with a ketone body

In our previous study we observed that hydroxybutyric acids were key metabolite biomarkers for ovarian cancer [9]. As the present and previous study were performed from the same serum samples, in patients with malignant disease we correlated 3-hydroxybutyric acid, a ketone body, with all the analyzed lipids in order to understand if the lipidomic alterations could result from fatty acid oxidation in the tumor cells. Indeed, especially PC and short chain TAG molecules showed negative correlation with serum 3-hydroxybutyric acid (Supplementary Table 7 and Figure 3C), suggesting that these lipids are consumed in tumors that are producing the ketone bodies. Among the most significantly correlating lipids, a positive correlation was observed only for a long chain TAG and acylcarnitine molecules.

### Lipid metabolism gene expression analyses

Our final aim was to investigate which lipid metabolism-related genes associate with overall survival of the ovarian cancer patients. To this end we analyzed 174 genes from relevant KEGG pathways and used TCGA data to investigate the association of expression and copy number with survival. A gene was considered significant if it had consistent association with survival both in the TCGA gene expression data (KM curves based on quartiles) and data obtained from online data analysis tool [10] (median split). With these criteria, only six genes were significant: low expression of *ABCD1*, *CEL*, *LPIN2* and *PLA2G2D* genes and high expression of *ADH1B* and *ASAH1* genes were associated with poor survival of the patients (Table 2, Supplementary Figure 3). These genes encode proteins related to sphingolipid metabolism, fatty acid import and oxidation and glycero(phospho)lipid metabolism.

**Table 2:** Results for lipid metabolism genes that showed significant association with survival both in the TCGA and KMplot.com data sets

Gene	TCGA *p*-value	KMplot.com *p*-value	Expression poor survival	CNA *p*-value	Enzyme	KEGG pathway
*ABCD1*	0.043	0.010	low	NA	ATP-Binding Cassette, Sub-Family D (ALD), Member 1	hsa04146: peroxisome
*ADH1B*	0.027	<0.001	high	0.368	Alcohol Dehydrogenase 1B (Class I), Beta Polypeptide	hsa00071: fatty acid degradation
*ASAH1*	0.003	0.007	high	0.067	N-Acylsphingosine Amidohydrolase (Acid Ceramidase) 1	hsa00600: sphingolipid metabolism
*CEL*	0.013	0.022	low	0.397	Carboxyl ester lipase	hsa00561: glycerolipid metabolism
*LPIN2*	0.002	0.042	low	0.326	Lipin 2	hsa00561: glycerolipid metabolism; hsa00564: glycerophospholipid metabolism
*PLA2G2D*	0.002	0.003	low	0.304	Phospholipase A2, Group IID	hsa00564: glycerophospholipid metabolism

Also the *p*-values from the copy number analyses (CNA) are shown.

## DISCUSSION

In this study we characterized the lipidome of serum and tumor samples from ovarian high-grade serous carcinoma patients. An overall decrease of lipid levels of most of the analyzed lipid classes was observed in ovarian cancer patient serum samples as compared to the controls. Our metabolomics analyses performed using the same sample series did not reveal such a systematic decrease [9], and therefore this phenomenon seems to be specific to lipid metabolism. Our data is consistent with other recent reports that have shown decrease of TAGs [11] and glycerophospholipids [12] in ovarian cancer patients. The global decrease of lipids can be at least partly explained by altered lipoprotein levels. For instance, HDL particles are rich in phospholipids and decreased HDL-cholesterol [13] and Apolipoprotein A-I (ApoAI) [14] levels have been reported in ovarian cancer patients. For other lipoproteins, LDL and VLDL, the direction of change in ovarian cancer patients is less clear [13, 15]. A limitation of our study was that we did not have clinical cholesterol measurements available from the samples, and therefore further lipoprotein and lipidomic studies are needed to understand the in-depth alterations of lipoprotein lipids in ovarian cancer patients. Another limitation was that data for BMI, a potential factor affecting the lipidome, was available for only 26 patients, and results from more extensive studies are needed to confirm our current findings which did not show association of ovarian cancer affected lipids with BMI.

Despite the global decrease of lipids, there were also exceptions showing consistent increase due to ovarian cancer, and especially in patients with more advanced disease. This was particularly prominent for ceramides, where the 16:0, 18:0, 20:0 and 24:1 FA containing ceramides were increased, while ceramides with 23:0 and 24:0 FAs were decreased. This pattern is strikingly similar with coronary artery disease patients having high risk of cardiovascular death, as we have shown that higher Cer(d18:1/16:0), Cer(d18:1/18:0), Cer(d18:1/20:0) and Cer(d18:1/24:1) ceramides predict increased risk, while Cer(d18:1/24:0) is a protective lipid for cardiovascular events [16, 17]. Indeed, ovarian cancer patients are reported to be at an increased risk of developing ischemic stroke [18], which may be even first sign of the disease [19]. This phenomenon could be explained by paraneoplastic thrombocytosis, as malignant ovarian tumors have been shown to produce cytokines promoting platelet production, which then induce tumor growth [20]. However, it is tempting to speculate that the alteration of ceramide profile may be also involved in the development of increased risk for ischemic strokes.

Among other lipids, SMs showed correlation between tumor tissue and serum samples, and SMs were also elevated in patients with incomplete tumor reduction and poorer survival. It is plausible that the increased ceramide levels could originate from conversion of SMs to ceramides in the tumor tissue, although our gene expression analyses did not show association of any the sphingomyelinase genes to overall survival. The only significant gene regarding sphingolipid metabolism was acid ceramidase (*ASAH1*), which catalyzes the hydrolysis of ceramides to produce sphingosine, which can be subsequently phosphorylated to S1P [21]. Increase of S1P levels have been reported in ascites [22] and plasma [23] of ovarian cancer patients, and it would be logical to assume that accumulation of ceramides would lead to conversion into S1P by the acid ceramidase. S1P is known to be potential modulator of several tumorigenic processes in ovarian cancer, including promotion of invasion, migration and proliferation of cancer cells as well as participation to hypoxic, angiogenetic and inflammatory processes [24]. Surprisingly in our data instead of increase we recorded a decrease of S1P lipids in the serum of ovarian cancer patients, which was not supporting the concept of ceramide conversion to S1P. This is in contrast with previous data in plasma, and a confirmation study is needed to resolve whether ovarian cancer patients exhibit decrease or increase of S1P in their serum.

Another interesting finding was the decrease of TAGs with shorter/medium chain fatty acyl side chains in cancer patients, whereas the longer chain ones were not altered or showed an increase. We observed that 3-hydroxybutyric acid negatively correlated especially with the TAGs with shorter fatty acyl side chains. This indirectly suggests that the fatty acyl side chains from TAGs are used in fatty acid oxidation producing ketone bodies. A possible reason for the different behavior of the longer fatty acyl side chains may stem from our gene expression findings. Low expression of *ABCD1*, the gene encoding for adrenoleukodystrophy protein (ALDP) was associated with worse overall survival. This protein is associated with transport of very-long-chain fatty acids into peroxisome for beta-oxidation [25]. Thus, it is possible that peroxisomal fatty acid oxidation is impaired. This is supported also by our previous metabolomics data which showed increase of metabolites that are found in peroxisomal disorder [9].

In summary, our analyses revealed that several lipid species, including ceramide, LPC, PC, SM and TAG lipid classes, showed both diagnostic and prognostic potential. Thus, it appears that the lipidomic alterations caused by ovarian cancer are profound and there are several potential candidates for further biomarker development. Lipids showed especially good performance in prognostic setting, and taken into account our previous data [9] it is reasonable to assume that measuring lipids and hydrophilic metabolites simultaneously would form a test with both diagnostic and prognostic value. Further research is needed to validate the most promising markers in independent cohorts and especially for early-stage patients that are not diagnosed with CA-125 measurements. Finally, mechanistic understanding behind the alterations may lead to potential therapeutic targets and possibilities for companion diagnostics.

## MATERIALS AND METHODS

### Patients and samples

Lipidomic profiling was performed for serum samples of 98 subjects without malignant disease, i.e. control group, as well as for 147 ovarian high-grade serous carcinoma patient serum samples. Clinicopathological characteristics of the study are shown in Supplementary Table 1. Before lipidomic analyses, cancer patient and control group samples were combined into one set and randomized. Lipidomic profiling was also performed for tumor tissues obtained from 140 patients having matching serum samples in the study.

We have recently described the collection and preparation of the samples in detail [9]. In brief, all tissue and serum samples were from preoperative primary ovarian cancer patients and collected at the Tumor Bank Ovarian Cancer (www.toc-network.de) at the Charité Medical University (Berlin, Germany) between 09/2000 and 02/2011. The Ethics Committee approved the use of the samples for the study, and the patient’s informed consent was obtained prior sample collection and documentation of clinical and surgical data. The study population without ovarian cancer consisted of a group of pelvic mass patients with benign tumors, endometriosis, uterus myomatosus, adnexitis and other conditions.

### Lipidomic analysis of serum samples (LC-MS/MS)

Lipidomic analyses were performed using two platforms, a global screening method and a phosphosphingolipid platform. Lipids for the screening method were extracted using a modified Folch extraction [26] and protein precipitation in methanol was used for the extraction of phosphosphingolipids. Prior to extraction, samples were thawed at +4°C, and Hamilton MICROLAB STAR system (Hamilton Robotics, Switzerland) was used for the extraction. For the screening method, samples (10 µl) were aliquoted into a 96-well plate, and internal standard mixture (20 µl) containing a known amount of synthetic internal standards (IS) was added followed by chloroform (450 µl). Organic phase separation was facilitated by adding 20 mM acetic acid and centrifuging the plate for 5 min at 500 × g. The lower organic phase (360 µl) was transferred into a new 96-well plate. The remaining water-containing phase was washed with additional chloroform (360 µl) followed by centrifugation and removal of the remaining organic phase. The two organic phases were pooled and evaporated under N2 until dryness. The lipid extracts were then re-dissolved in chloroform:methanol (1:2, v/v). For the analysis of phosphosphingolipids, samples (25 µl) were aliquoted into a 96-well plate, and ice-cold methanol containing 0.1% BHT (500 µl) was added to each sample, followed by internal standard mixture (25 µl) containing a known amount of synthetic standards. Samples were mixed and incubated for 10 min. After centrifugation, supernatant (450 µl) was transferred into a new 96-well plate, evaporated under N2 until dryness and re-dissolved in methanol (200 µL).

Lipidomics screening and phosphosphingolipid platforms were both analyzed on a hybrid triple quadrupole/linear ion trap mass spectrometer (QTRAP 5500, AB Sciex, Concors, Canada) equipped with an ultra-high performance liquid chromatography (UHPLC) (Nexera-X2, Shimadzu). Chromatographic separation of the lipidomics screening platform was performed on Acquity BEH C18, 2.1 × 50 mm id. 1.7 µm column (Waters, Massachusetts, USA). Mobile phases consisted of (A) 10 mM ammonium acetate in LC-MS grade water with 0.1% formic acid, and (B) 10 mM ammonium acetate in acetonitrile:2-propanol (3:4, V/V) with 0.1% formic acid (FA). The following LC gradient was used: 0.3 min at 45% B, linear increase of B from 45% to 95% in 10 min, 95% to 100% B in 0.1 min, 2.5 min at 100% B, 100% to 45% B in 0.1 min and 1.5 min equilibration at 45% prior to the next injection. Flow rate was 600 µl/min and column temperature 60°C. Injection volume of all samples was 2 µl. Chromatographic separation of phosphosphingolipid platform was performed on AQUASIL C18, 2.1 × 50 mm, 5 µm (Thermo Fisher, Massachusetts, USA), column set at 60°C. Mobile phases consisted of (A) 10 mM ammonium acetate in LC-MS grade water with 0.1% formic acid, and (B) 10 mM ammonium acetate in methanol:2-Propanol (1:2) with 0.1% formic acid. Flow rate was 1000 µl/min and sample volume 5 µl. First, solvent B was kept at 20% for 1 min, then linearly increased to 100% in 4 min. The column was flushed with 100% B for 3 min, followed by 2 min equilibration at 20% B.

For the MS analysis, a targeted approach in positive ion mode was used for both platforms. Data was collected using scheduled multiple reaction monitoring (sMRM™) algorithm for the lipidomics screening platform [27] and multiple reaction monitoring (MRM) for phosphosphingolipids. Mass spectrometer parameters were optimized based on lipid class. Lipidomics data were processed using Analyst and MultiQuant 3.0 software (QTRAP 5500, AB Sciex, Concors, Canada), area or height ratios of analyte and its corresponding IS peak were normalized with IS amount and sample volume.

Only those lipids were subjected for statistical analyses that were found in at least 90% of all the samples.

### Lipidomic analysis of tumor samples (LC-MS)

Lipidomic analysis of tumor samples was performed with a UPLC-MS-QTOF method, and in addition to the previously described LC-MS/MS method, this method was also used to analyze the serum samples so that correlation of lipids between tumor and serum was possible to perform. The details of this method have been described in Supplementary Materials and Methods.

### Statistical analyses

All statistical analyses were performed using R, version x64 3.2.3. After log-transformation of the data, two group comparisons were performed by unpaired *t*-tests and calculating mean relative differences between the groups. For multiple group comparisons Analysis of Variance (ANOVA) was performed. Correlation analyses were performed by Pearson or Spearman method, as appropriate and specified in each case. Multiple hypothesis correction was evaluated by false discovery rate *q*-values. The results presented in the main text show unadjusted *p-*values, and *p-*values together with *q*-values are presented for all analyses in Supplementary Table 2. Association of the lipids to survival was investigated by cox proportional hazards regression models and Kaplan-Meier plots with a median split and logrank test (package *survival*). For cox regression models, the data was log-transformed and divided by standard deviation. Multivariable models were adjusted with age and incomplete/complete tumor reduction in surgery. For overall survival, also a model with additional adjustment of tumor stage was constructed. Age-adjusted *p-*values in malignant vs. benign comparison were obtained by logistic regression model incorporating both lipid and age in the model. Logistic regression models were also used to estimate the combined AUC values for lipids and CA-125, and the AUC values were calculated using *ROCR* library [28]. Heatmaps were visualized with Tableau software, version 10.1.1.

Survival analyses on those genes that were related to lipids with diagnostic or prognostic potential was performed based on gene expression and copy number data. The KEGG genes were selected from the following KEGG pathways: fatty acid degradation, sphingolipid metabolism, glycerolipid metabolism, glycerophospholipid metabolism and peroxisome (only lipid metabolism related genes). TCGA mRNA expression and clinical information for ovarian cancer patient samples were obtained from TCGA data portal [29]. Copy number alterations for tumors were obtained from cBioPortal [30]. When interpreting the results, the significance of the results was evaluated by both *p-*value and investigating the KM curves manually. Another independent data set and online tool was used to analyze the association of gene expression with survival in ovarian cancer patients (survival = overall survival, histology = serous carcinoma, follow up threshold = 10 years) [10]. In this data set those probes were selected that showed most significant *p-*value for each gene.

## SUPPLEMENTARY MATERIALS FIGURES AND TABLES













## Abbreviations

ApoAI: Apolipoprotein A-I
CE: cholesterylester
Cer: ceramide
DAG: diacylglycerol
FA: fatty acid
Gb3: globotriasoylceramide
Glc/GalCer: glucosyl/galactosylceramide
IS: internal standard
LacCer: lactosylceramide
LC: liquid chromatography
LC-MS: liquid chromatography-mass spectrometry
LC-MS/MS: liquid chromatography-tandem mass spectrometry
LPC: lysophosphatidylcholine
LPE: lysophosphatidylethanolamine
NMR: nuclear magnetic resonance
MRM: multiple reaction monitoring
MS: mass spectrometry
PC: phosphatidylcholine
PC O: alkyl-linked phosphatidylcholine
PC P: alkenyl-linked phosphatidylcholine
PE: phosphatidylethanolamine
PE O: alkyl-linked phosphatidylethanolamine
PE P: alkenyl-linked phosphatidylethanolamine
PI: phosphatidylinositol
S1P: sphingosine-1-phosphate
SM: sphingomyelin
TAG: triacylglycerol
UPLC: ultra performance liquid chromatography
