# Supplementary material for: High-grade ovarian serous carcinoma patients exhibit profound alterations in lipid metabolism
Source: Oncotarget. 2017 Oct 26;8(61):102912–22. doi: 10.18632/oncotarget.22076 (PMC5732699; doi:10.18632/oncotarget.22076)
Supplement: Supplementary file 1 [file oncotarget-08-102912-s001.pdf]

High-grade ovarian serous carcinoma patients exhibit profound alterations in lipid metabolism

SUPPLEMENTARY MATERIALS

Lipidomic analysis of tumor samples (LC-MS)

The lipidomics data with the method described previously [1] and in this section was used in the tumor tissue analyses and correlation analyses of tumor tissue and serum. Tumor samples were prepared by homogenizing 2.5 mg of tissue with Retsch homogenizer (3 min, 20 Hz). The samples were homogenized into 0.9% NaCl, and the volume was adjusted to get concentration of approximately 0.05 mg/µl. The samples (50 µl of homogenate) were extracted with a one-step chloroform:methanol (2:1; 200 µl) protocol after adding an internal standard mixture with Cer(d18:1/17:0), PC(17:0/0:0), PC(17:0/17:0), PE(17:0/17:0) and TG(17:0/17:0/17:0) at concentration level of 0.5–1 µg/sample. The samples were vortexed for 2 min, incubated 30 min at RT and centrifuged at 7800 g for 3 min. A labelled lipid standard mixture containing PC(16:0-D_3/0:0), PC(16:0/16:0-D_6) and TG(16:0/16:0/16:0-$^{13}C_3$) at concentration level of 0.5 µg/sample was added into the lower chloroform phase (100 µl) before UPLC-MS analysis.

10 µl of serum samples were used for the analyses, and the samples were prepared by adding an internal standard mixture with DG(17:0/17:0/0:0), CE(19:0), Cer(d18:1/17:0), MG(17:0/0:0/0:0), PA(17:0/17:0), PC(17:0/0:0), PC(17:0/17:0), PE(17:0/17:0), PG(17:0/17:0) and TG(17:0/17:0/17:0) at concentration level of 0.2–1.6 µg/sample and extracting with chloroform:methanol (2:1, 100 µl), as for the tumor samples. The samples were vortexed for 2 min, incubated 30 min at RT and centrifuged at 7800g for 3 min. A labelled lipid standard mixture containing PC(16:0-D_3/0:0), PC(16:0/16:0-D_6) and TG(16:0/16:0/16:0-13C_3) at concentration level of 0.1–0.15 µg/sample was added into the separated lipid extracts (60 µl) before UPLC-MS analysis.

Lipid extracts from tumor and serum samples were analyzed on a Waters Q-Tof Premier mass spectrometer combined with an Acquity Ultra Performance LC™ (UPLC™). The column was an Acquity UPLC™ BEH C18 2.1 × 100 mm with 1.7 µm particles. The solvent system included A) water (1% 1 M NH_4Ac, 0.1% HCOOH) and B) acetonitrile/isopropanol (1:1, 1% 1M NH_4Ac, 0.1% HCOOH). The gradient started from 65% A / 35% B, reached 80% B in 2 min, 100% B in 7 min and remained there for the next 7 min. There was a 4 min re-equilibration step before the following run. The flow rate was 0.400 ml/min and the injected amount 1.0 µl. Reserpine was used as the lock spray reference compound. The lipid profiling was performed using ESI in positive mode, and the data were collected at a mass range of m/z 300–1200 with scan duration of 0.2 sec.

The data were processed using MZmine 2 software [2] and the processing included alignment of peaks, peak integration, normalization, and peak identification. Lipid identification was based on an internal spectral library. The data were normalized using internal standard representatives of each class of lipid present in the samples: the intensity of each identified lipid was normalized by dividing it with the intensity of its corresponding standard and multiplying it by the concentration of the standard. Other molecular species (e.g. SM and unidentified lipids) were normalized with PC (17:0/0:0) for retention time < 300 s, PC (17:0/17:0) for retention time between 300 and 410 s, and TG (17:0/17:0/17:0) for higher retention times. The data from tumor samples were normalized with sample weight. Zero values in the data were imputed with a value corresponding to the half of the minimum value of the corresponding lipid across all samples.

REFERENCES

1. Nygren H, Seppänen-Laakso T, Castillo S, Hyötyläinen T, Oresic M. Liquid Chromatography-Mass Spectrometry (LC-MS)-Based Lipidomics for Studies of Body Fluids and Tissues. Methods Mol Biol. 2011; 708:247–57. https://doi.org/10.1007/978-1-61737-985-7_15.
2. Pluskal T, Castillo S, Villar-Briones A, Oresic M. MZmine 2: modular framework for processing, visualizing, and analyzing mass spectrometry-based molecular profile data. BMC Bioinformatics. 2010; 11:395. https://doi.org/10.1186/1471-2105-11-395.

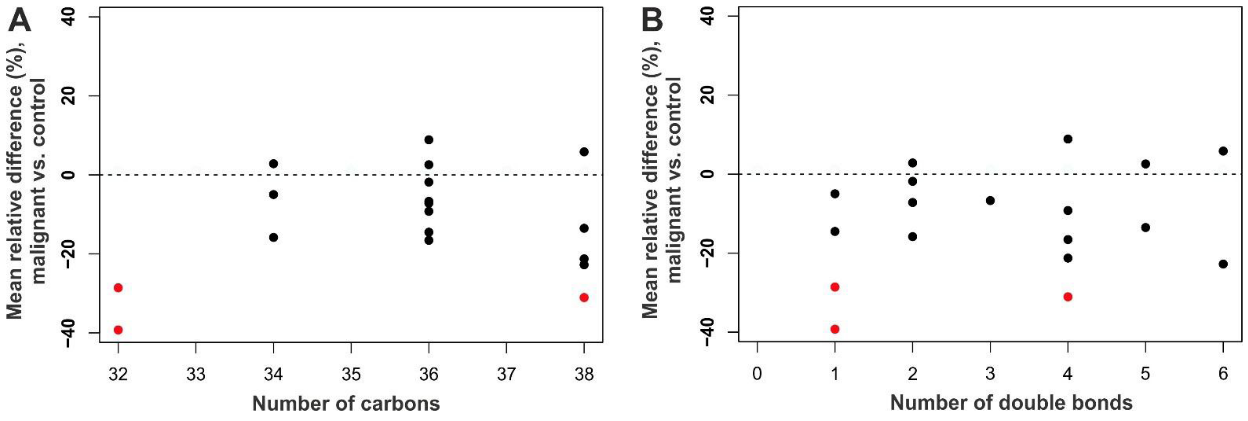

Supplementary Figure 1: (**A**) Mean relative difference of diacylglycerol (DAG) lipids according to the total number of carbons in the FA side chains. (**B**) Mean relative difference of DAG lipids according to the total number of double bonds in the FA side chains. Red color indicates statistically significant ($p < 0.05$) result.

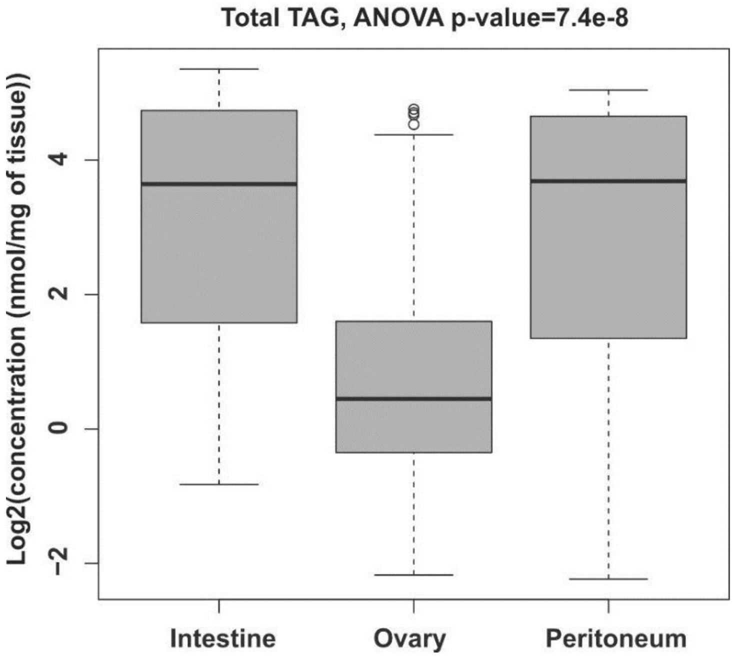

Supplementary Figure 2: Level of total triacylglycerols (TAGs) in tumor samples obtained from intestine, ovaries or peritoneum.

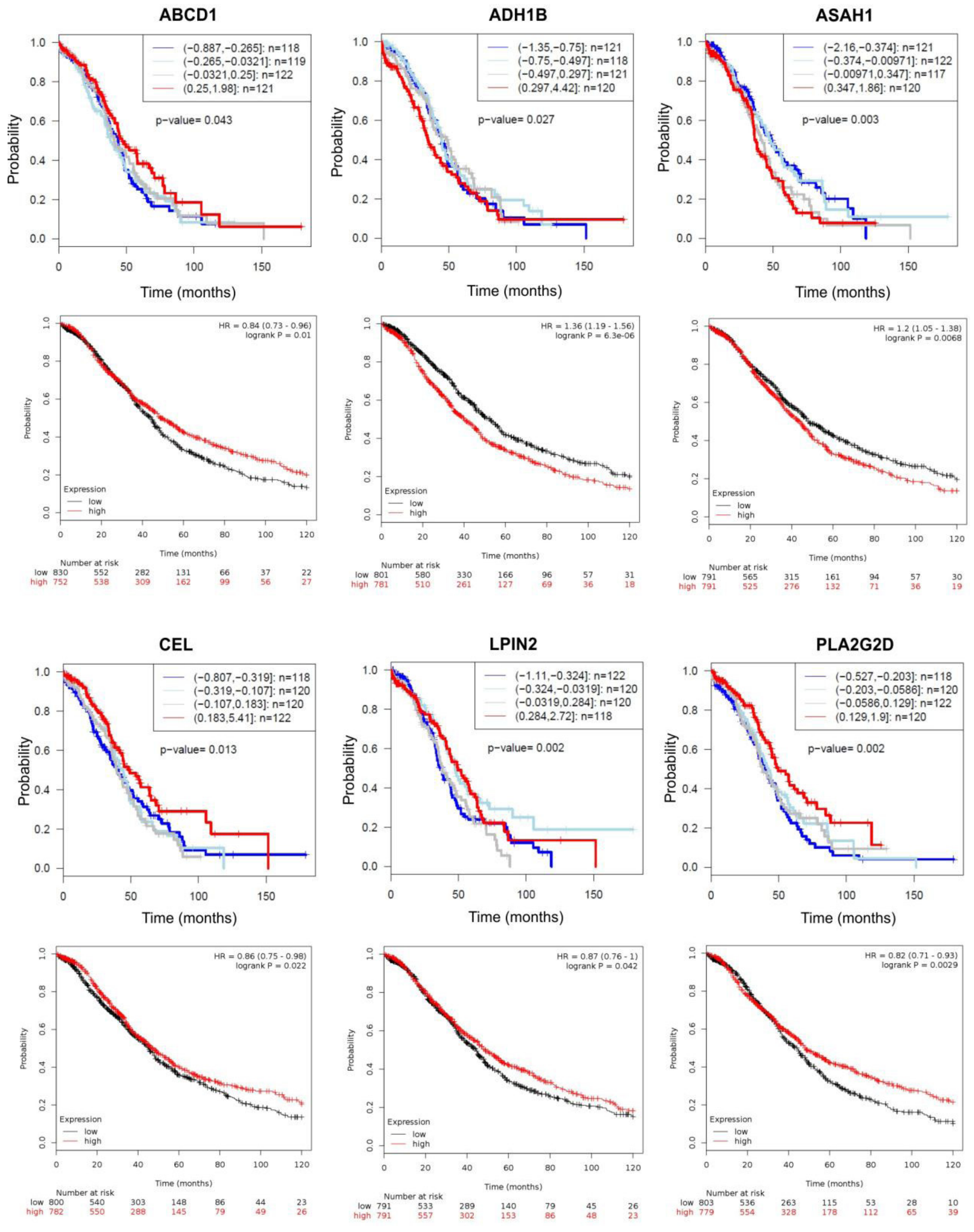

Supplementary Figure 3: Overall survival data for significant genes in TCGA data (upper panels) and KMplot.com (lower panels).

Supplementary Table 1: Clinicopathological characteristics of the study cohort

		Serum samples		Tumor samples	
		Cases	%	Cases	%
**All patients**					
Malignancy	malignant cases	147	60%	140	-
	non-malignant cases	98	40%	-	-
**Patients with malignant tumors**					
FIGO stage	I	2	1%	2	1%
	II	5	3%	4	3%
	III	99	67%	96	69%
	IV	31	21%	27	19%
	NA	10	7%	11	8%
Residual tumor mass in surgery	complete reduction	89	61%	83	59%
	<0.5 cm	30	20%	31	22%
	<1 cm	20	14%	19	14%
	<2 cm	2	1%	2	1%
	>2 cm	6	4%	5	4%
Location of analyzed tumor sample	ovary	-	-	73	52%
	intestine	-	-	20	14%
	peritoneum	-	-	16	11%
	other	-	-	29	21%
	NA	-	-	2	1%
Ascites	no	29	20%	27	19%
	<500 mL	54	37%	53	38%
	>500 mL	64	44%	60	43%
Progress-free survival	event	87	59%	77	55%
	median follow-up (months)	16	-	17	-
	no event	60	41%	63	45%
	median follow-up (months)	26		21	-
Overall survival	event	80	54%	79	56%
	median follow-up (months)	26		25	
	no event	67	46%	61	44%
	median follow-up (months)	47		48	

Supplementary Table 2: Results of all statistical analyses for serum lipids. See Supplementary_Table_2

Supplementary Table 3: Lipids that showed consistent direction of alteration in patients with incomplete vs. complete tumor reduction during surgery as well in comparison of patients with malignant vs. benign condition. See Supplementary_Table_3

Supplementary Table 4: Overall survival results incorporating also stage of the disease in the cox regression model (right column)

LIPID NAME	LIPID CLASS	UNIVARIATE			MULTIVARIATE (age, tumor reduction)			MULTIVARIATE (age, tumor reduction, stage)		
		**UHR (95% CI)**	***p*-value**	***q*-value**	**MHR (95% CI)**	***p*-value**	***q*-value**	**MHR (95% CI)**	***p*-value**	***q*-value**
CE 14:1	CE	0.77 (0.61, 0.98)	**0.033**	0.107	0.77 (0.60, 0.99)	**0.038**	0.256	0.81 (0.63, 1.04)	0.094	0.521
CE 17:0	CE	0.77 (0.63, 0.95)	**0.013**	0.074	0.80 (0.64, 0.99)	**0.043**	0.262	0.87 (0.69, 1.09)	0.220	0.585
CE 22:3	CE	0.75 (0.59, 0.94)	**0.012**	0.073	0.79 (0.63, 0.98)	**0.034**	0.252	0.90 (0.72, 1.13)	0.360	0.625
Cer(d16:1/23:0)	Cer d16:1	0.76 (0.61, 0.95)	**0.015**	0.075	0.78 (0.62, 0.97)	**0.028**	0.252	0.80 (0.63, 1.01)	0.060	0.521
Cer(d18:1/16:0)	Cer d18:1	1.47 (1.15, 1.87)	**0.002**	**0.038**	1.36 (1.06, 1.75)	**0.014**	0.252	1.22 (0.95, 1.57)	0.120	0.521
Cer(d20:1/24:1)	Cer d20:1	1.54 (1.21, 1.97)	**0.001**	**0.026**	1.32 (1.03, 1.71)	**0.031**	0.252	1.09 (0.84, 1.43)	0.510	0.691
LPC 14:0_sn1	LPC	0.72 (0.57, 0.90)	**0.004**	**0.048**	0.79 (0.62, 1.00)	**0.046**	0.263	0.77 (0.60, 0.98)	**0.033**	0.521
LPC 18:2_sn1	LPC	0.67 (0.52, 0.84)	**0.001**	**0.026**	0.75 (0.58, 0.97)	**0.027**	0.252	0.79 (0.60, 1.03)	0.080	0.521
LPC 18:2_sn2	LPC	0.67 (0.53, 0.85)	**0.001**	**0.026**	0.75 (0.58, 0.96)	**0.021**	0.252	0.78 (0.60, 1.01)	0.063	0.521
LPC 20:0_sn2	LPC	0.69 (0.54, 0.88)	**0.003**	**0.048**	0.76 (0.59, 0.98)	**0.035**	0.252	0.76 (0.59, 1.00)	**0.047**	0.521
LPC 20:2_sn1	LPC	0.73 (0.58, 0.91)	**0.004**	**0.048**	0.78 (0.62, 0.98)	**0.031**	0.252	0.76 (0.60, 0.98)	**0.031**	0.521
LPC 20:2_sn2	LPC	0.72 (0.58, 0.90)	**0.004**	**0.048**	0.78 (0.62, 0.98)	**0.031**	0.252	0.75 (0.58, 0.97)	**0.026**	0.521
LPC 22:0_sn1	LPC	0.70 (0.55, 0.89)	**0.004**	**0.048**	0.78 (0.61, 0.99)	**0.044**	0.263	0.81 (0.63, 1.04)	0.100	0.521
LPC 24:0_sn2	LPC	0.65 (0.50, 0.84)	**0.001**	**0.033**	0.70 (0.54, 0.91)	**0.008**	0.200	0.81 (0.62, 1.07)	0.130	0.528
LPE 18:2_sn2	LPE	0.68 (0.53, 0.88)	**0.004**	**0.048**	0.72 (0.56, 0.94)	**0.016**	0.252	0.73 (0.56, 0.96)	**0.023**	0.521
PC 34:3b	PC	0.74 (0.59, 0.94)	**0.014**	0.074	0.79 (0.63, 0.99)	**0.042**	0.261	0.79 (0.62, 1.01)	0.056	0.521
PC 34:5	PC	0.67 (0.51, 0.88)	**0.004**	**0.048**	0.68 (0.52, 0.90)	**0.006**	0.187	0.75 (0.58, 0.97)	**0.031**	0.521
PC 35:0	PC	0.74 (0.59, 0.92)	**0.008**	0.065	0.74 (0.59, 0.92)	**0.007**	0.193	0.77 (0.61, 0.98)	**0.031**	0.521
PC 35:2b	PC	0.79 (0.63, 1.00)	**0.049**	0.126	0.79 (0.63, 0.98)	**0.035**	0.252	0.80 (0.63, 1.01)	0.063	0.521
PC 35:3a	PC	0.75 (0.59, 0.96)	**0.024**	0.095	0.79 (0.62, 1.00)	**0.047**	0.263	0.82 (0.64, 1.06)	0.130	0.528
PC 37:3	PC	0.74 (0.60, 0.93)	**0.009**	0.066	0.79 (0.64, 0.97)	**0.025**	0.252	0.79 (0.63, 0.99)	**0.039**	0.521
PC 38:6a	PC	0.73 (0.58, 0.92)	**0.007**	0.061	0.80 (0.64, 1.00)	**0.046**	0.263	0.81 (0.65, 1.02)	0.077	0.521
PC O-36:1	PC O	0.76 (0.62, 0.94)	**0.011**	0.072	0.78 (0.63, 0.97)	**0.028**	0.252	0.81 (0.64, 1.01)	0.064	0.521
PC O-38:1	PC O	0.71 (0.57, 0.88)	**0.002**	**0.036**	0.77 (0.63, 0.96)	**0.017**	0.252	0.80 (0.64, 1.01)	0.059	0.521
PC O-38:2	PC O	0.65 (0.51, 0.82)	**0.000**	**0.026**	0.70 (0.56, 0.88)	**0.002**	0.164	0.74 (0.58, 0.94)	**0.013**	0.521
PI 32:0	PI	0.70 (0.51, 0.98)	**0.037**	0.110	0.70 (0.50, 0.98)	**0.036**	0.253	0.72 (0.53, 0.98)	**0.040**	0.521
SM 41:1	SM	0.70 (0.58, 0.86)	**0.001**	**0.026**	0.78 (0.63, 0.96)	**0.019**	0.252	0.80 (0.64, 1.01)	0.059	0.521
TAG(18:1/18:1/20:4)	TAG	1.38 (1.10, 1.74)	**0.006**	0.052	1.32 (1.03, 1.69)	**0.026**	0.252	1.20 (0.93, 1.55)	0.160	0.559

Supplementary Table 5: Cox regression models for lipids measured from tumor tissue samples. e, ether-linked phospholipid. See Supplementary_Table_5

Supplementary Table 6: Correlation of lipids in serum and tumor tissue samples. See Supplementary_Table_6

Supplementary Table 7: Results for lipids that were most significantly correlating ($p < 0.001$) with 3-hydroxybutyric acid in ovarian cancer patient serum samples. See Supplementary_Table_7
