# Supplementary material for: High-grade ovarian serous carcinoma patients exhibit profound alterations in lipid metabolism
Source: Oncotarget. 2017 Oct 26;8(61):102912–22. doi: 10.18632/oncotarget.22076 (PMC5732699; doi:10.18632/oncotarget.22076)
Supplement: Supplementary file 3 [file oncotarget-08-102912-s003.docx]

Supplementary Table 3**: Lipids that showed consistent direction of alteration in patients with incomplete vs. complete tumor reduction during surgery as well in comparison of patients with malignant vs. benign condition**

|  |  | **Incomplete vs. complete tumor reduction** | | | **Malignant vs. benign** | | |
| --- | --- | --- | --- | --- | --- | --- | --- |
| **LIPID NAME** | **LIPID CLASS** | **Difference (%)** | **p-value** | **q-value** | **Difference (%)** | **p-value** | **q-value** |
| CE 14:0 | CE | -13.6 | 0.049 | 0.135 | -23.2 | 2.0E-06 | 2.1E-06 |
| CE 15:0 | CE | -14.8 | 0.043 | 0.127 | -17.4 | 1.4E-04 | 1.0E-04 |
| CE 17:0 | CE | -18.8 | 0.004 | 0.075 | -17.1 | 7.7E-05 | 5.9E-05 |
| CE 18:0 | CE | -16.5 | 0.013 | 0.081 | -27.2 | 3.3E-10 | 8.9E-10 |
| CE 18:3 | CE | -17.2 | 0.009 | 0.079 | -24.1 | 1.4E-10 | 4.1E-10 |
| CE 20:2 | CE | -13.9 | 0.044 | 0.129 | -24.1 | 1.3E-07 | 1.8E-07 |
| CE 20:3 | CE | -13.2 | 0.046 | 0.132 | -21.7 | 4.5E-06 | 4.5E-06 |
| CE 20:4 | CE | -11.0 | 0.037 | 0.126 | -7.7 | 0.043 | 0.020 |
| CE 22:3 | CE | -23.5 | 0.013 | 0.081 | -15.2 | 0.049 | 0.023 |
| Cer(d16:1/23:0) | Cer d16:1 | -19.4 | 0.013 | 0.081 | -26.2 | 2.8E-04 | 1.9E-04 |
| Cer(d16:1/24:0) | Cer d16:1 | -15.0 | 0.012 | 0.081 | -30.4 | 2.4E-08 | 4.1E-08 |
| Cer(d18:0/18:0) | Cer d18:0 | 38.2 | 0.006 | 0.075 | 50.0 | 7.3E-05 | 5.7E-05 |
| Cer(d18:0/24:1) | Cer d18:0 | 22.5 | 0.014 | 0.084 | 15.2 | 0.023 | 0.012 |
| Cer(d18:1/16:0) | Cer d18:1 | 15.7 | 0.006 | 0.075 | 27.3 | 1.4E-05 | 1.3E-05 |
| Cer(d18:1/18:0) | Cer d18:1 | 26.0 | 0.004 | 0.075 | 72.2 | 1.0E-11 | 4.0E-11 |
| Cer(d18:1/20:0) | Cer d18:1 | 18.4 | 0.008 | 0.075 | 39.8 | 1.8E-07 | 2.4E-07 |
| Cer(d18:1/24:1) | Cer d18:1 | 17.4 | 0.012 | 0.081 | 29.9 | 2.4E-06 | 2.4E-06 |
| Cer(d18:2/23:0) | Cer d18:2 | -18.3 | 0.007 | 0.075 | -18.9 | 4.1E-04 | 2.8E-04 |
| Cer(d18:2/24:0) | Cer d18:2 | -12.5 | 0.023 | 0.104 | -23.0 | 6.2E-06 | 6.0E-06 |
| Cer(d18:2/26:0) | Cer d18:2 | -16.3 | 0.016 | 0.090 | -14.4 | 0.037 | 0.018 |
| Cer(d20:1/22:0) | Cer d20:1 | 31.6 | 0.001 | 0.075 | 22.0 | 0.003 | 0.002 |
| Cer(d20:1/24:1) | Cer d20:1 | 42.4 | 1.2E-04 | 0.033 | 43.6 | 4.2E-07 | 5.1E-07 |
| Glc/GalCer(d16:1/23:0) | Glc/GalCer | -20.6 | 0.012 | 0.081 | -20.7 | 1.9E-04 | 1.4E-04 |
| Glc/GalCer(d18:1/26:0) | Glc/GalCer | -12.4 | 0.045 | 0.132 | -21.3 | 7.6E-05 | 5.9E-05 |
| Glc/GalCer(d18:2/23:0) | Glc/GalCer | -15.4 | 0.032 | 0.120 | -21.7 | 5.0E-05 | 4.1E-05 |
| LacCer(d18:1/23:0) | LacCer | -15.4 | 0.033 | 0.120 | -26.4 | 1.1E-07 | 1.6E-07 |
| LPC 14:0_sn2 | LPC | -17.6 | 0.030 | 0.119 | -30.9 | 1.4E-05 | 1.3E-05 |
| LPC 18:2_sn1 | LPC | -20.1 | 0.011 | 0.081 | -30.8 | 8.7E-08 | 1.3E-07 |
| LPC 18:2_sn2 | LPC | -16.7 | 0.027 | 0.112 | -35.9 | 6.4E-13 | 3.5E-12 |
| LPC 20:0_sn1 | LPC | -19.5 | 0.007 | 0.075 | -16.5 | 0.004 | 0.002 |
| LPC 20:0_sn2 | LPC | -21.5 | 0.002 | 0.075 | -14.4 | 0.013 | 0.007 |
| LPC 20:2_sn2 | LPC | -16.9 | 0.038 | 0.126 | -17.0 | 2.1E-05 | 1.9E-05 |
| LPC 20:3_sn1 | LPC | -17.1 | 0.024 | 0.107 | -17.1 | 0.001 | 0.001 |
| LPC 22:0_sn1 | LPC | -15.5 | 0.008 | 0.075 | -28.8 | 7.4E-10 | 1.8E-09 |
| LPC 24:0_sn1 | LPC | -14.8 | 0.007 | 0.075 | -28.8 | 1.6E-12 | 8.4E-12 |
| LPC 24:0_sn2 | LPC | -13.2 | 0.011 | 0.081 | -29.1 | 2.5E-13 | 1.7E-12 |
| LPC O-20:0 | LPC O | -16.8 | 0.026 | 0.110 | -15.3 | 0.001 | 0.001 |
| LPC O-22:0 | LPC O | -12.4 | 0.036 | 0.126 | -25.3 | 6.2E-11 | 2.1E-10 |
| LPC O-22:1 | LPC O | -17.6 | 0.041 | 0.127 | -17.2 | 0.002 | 0.001 |
| PC 36:5a | PC | -13.9 | 0.022 | 0.104 | -33.1 | 5.7E-08 | 9.0E-08 |
| PC 37:1 | PC | -10.4 | 0.047 | 0.132 | -20.3 | 6.7E-06 | 6.3E-06 |
| PC 38:6a | PC | -18.6 | 0.007 | 0.075 | -35.9 | 8.0E-11 | 2.6E-10 |
| PC 38:6b | PC | -15.4 | 0.018 | 0.093 | -38.8 | 2.7E-13 | 1.7E-12 |
| PC 39:4 | PC | -13.3 | 0.023 | 0.104 | -19.1 | 9.9E-06 | 9.1E-06 |
| PC O-36:1 | PC O | -16.6 | 0.023 | 0.104 | -28.0 | 1.1E-07 | 1.6E-07 |
| PC O-38:1 | PC O | -12.1 | 0.032 | 0.120 | -28.5 | 2.9E-12 | 1.3E-11 |
| PC O-38:2 | PC O | -18.8 | 0.002 | 0.075 | -28.8 | 3.2E-09 | 6.6E-09 |
| PE O-34:1 | PE O | -17.7 | 0.032 | 0.120 | -13.1 | 0.041 | 0.020 |
| PE O-36:4 | PE O | -24.4 | 0.010 | 0.081 | -50.1 | 2.5E-11 | 9.5E-11 |
| PE O-38:4 | PE O | -17.1 | 0.036 | 0.126 | -49.4 | 1.1E-12 | 6.0E-12 |
| PE O-38:5 | PE O | -23.7 | 0.006 | 0.075 | -39.1 | 8.0E-10 | 1.9E-09 |
| PE O-38:6 | PE O | -18.5 | 0.026 | 0.110 | -15.9 | 0.047 | 0.022 |
| PE P-36:4 | PE O | -18.8 | 0.029 | 0.117 | -31.5 | 1.2E-06 | 1.3E-06 |
| S1P d16:1 | S1P | -8.6 | 0.040 | 0.126 | -20.5 | 6.4E-09 | 1.3E-08 |
| SM 30:2 | SM | -19.2 | 0.003 | 0.075 | -35.3 | 5.0E-11 | 1.7E-10 |
| SM 31:1 | SM | -17.9 | 0.008 | 0.075 | -24.9 | 1.2E-07 | 1.7E-07 |
| SM 32:1 | SM | -13.3 | 0.010 | 0.081 | -17.7 | 5.1E-06 | 5.0E-06 |
| SM 32:2 | SM | -18.4 | 0.005 | 0.075 | -27.8 | 5.8E-11 | 2.0E-10 |
| SM 39:1 | SM | -18.9 | 0.001 | 0.075 | -27.8 | 1.2E-10 | 3.6E-10 |
| SM 40:2b | SM | -14.6 | 0.009 | 0.079 | -27.2 | 7.1E-12 | 3.1E-11 |
| SM 41:1 | SM | -17.4 | 0.001 | 0.075 | -25.7 | 4.6E-12 | 2.0E-11 |
| SM 41:2a | SM | -17.0 | 0.004 | 0.075 | -17.2 | 7.1E-05 | 5.6E-05 |
| SM 42:1 | SM | -13.2 | 0.014 | 0.084 | -31.3 | 2.3E-13 | 1.6E-12 |
